# Exploratory Space-Time Analyses of Rift Valley Fever in South Africa in 2008–2011

**DOI:** 10.1371/journal.pntd.0001808

**Published:** 2012-08-28

**Authors:** Raphaëlle Métras, Thibaud Porphyre, Dirk U. Pfeiffer, Alan Kemp, Peter N. Thompson, Lisa M. Collins, Richard G. White

**Affiliations:** 1 Veterinary Epidemiology and Public Health Group, Department of Veterinary Clinical Sciences, Royal Veterinary College, Hatfield, United Kingdom; 2 Centre for the Mathematical Modelling of Infectious Diseases and Faculty of Infectious Disease Epidemiology, London School of Hygiene and Tropical Medicine, London, United Kingdom; 3 Epidemiology Group, Centre for Immunity, Infection and Evolution, University of Edinburgh, Ashworth Laboratories, Edinburgh, United Kingdom; 4 Centre for Emerging Zoonotic Diseases, National Institute for Communicable Diseases, National Health Laboratory Service, Sandringham, South Africa; 5 Epidemiology Section, Department of Production Animal Studies, University of Pretoria, Onderstepoort, South Africa; 6 School of Biological Sciences, Queen's University Belfast, Medical Biology Centre, Belfast, United Kingdom; Centers for Disease Control and Prevention, Kenya

## Abstract

**Background:**

Rift Valley fever (RVF) is a zoonotic arbovirosis for which the primary hosts are domestic livestock (cattle, sheep and goats). RVF was first described in South Africa in 1950–1951. Mechanisms for short and long distance transmission have been hypothesised, but there is little supporting evidence. Here we describe RVF occurrence and spatial distribution in South Africa in 2008–11, and investigate the presence of a contagious process in order to generate hypotheses on the different mechanisms of transmission.

**Methodology/Principal Findings:**

A total of 658 cases were extracted from World Animal Health Information Database. Descriptive statistics, epidemic curves and maps were produced. The space-time *K*-function was used to test for evidence of space-time interaction. Five RVF outbreak waves (one in 2008, two in 2009, one in 2010 and one in 2011) of varying duration, location and size were reported. About 70% of cases (n = 471) occurred in 2010, when the epidemic was almost country-wide. No strong evidence of space-time interaction was found for 2008 or the second wave in 2009. In the first wave of 2009, a significant space-time interaction was detected for up to one month and over 40 km. In 2010 and 2011 a significant intense, short and localised space-time interaction (up to 3 days and 15 km) was detected, followed by one of lower intensity (up to 2 weeks and 35 to 90 km).

**Conclusions/Significance:**

The description of the spatiotemporal patterns of RVF in South Africa between 2008 and 2011 supports the hypothesis that during an epidemic, disease spread may be supported by factors other than active vector dispersal. Limitations of under-reporting and space-time *K*-function properties are discussed. Further spatial analyses and data are required to explain factors and mechanisms driving RVF spread.

## Author Summary

The factors explaining Rift Valley fever (RVF) spread in domestic livestock during an epidemic are attributed to short and long distance mechanisms, including active vector dispersal, passive vector dispersal and movements of infectious animals. However, because of data scarcity, quantifying and disentangling these mechanisms remains challenging. Here, we generate hypotheses on the possible mechanisms involved in RVF spread in South Africa between 2008 and 2011. We use descriptive statistics and estimate the space-time K-function to explore the presence of space-time interactions, being interpreted as an indicator of an underlying transmission process. Our results confirm the presence of an intense, short, initial transmission process that could be attributed to active vector dispersal; but also highlight the presence of another transmission mechanism of a lower intensity and over further distances that could be explained by the movements of infectious animals, passive vector dispersal or emergence of other foci. Further data collection and modelling tools are required to confirm these hypotheses.

## Introduction

Rift Valley fever (RVF) is a vector-borne zoonotic disease caused by infection with a *Phlebovirus* (Family *Bunyaviridae*). The main vectors are mosquitoes from the genera *Aedes* and *Culex*; primary hosts are domestic livestock (cattle, sheep and goats), but the disease can also affect camels, buffaloes and other wild animals [1]. Since its first description in Kenya in 1931 [2], RVF has been reported in several African countries, and in the Arabian Peninsula [3]. Transmission to humans is mainly through contact with infectious animals or animal tissues, and symptoms vary from a flu-like illness to more severe conditions such as meningoencephalitis, haemorrhagic fever or death. In animals, RVF is of economic importance, causing waves of abortions at all stages of pregnancy and high mortality in newborn animals [1], [4].

Rift Valley fever epidemics have been reported following inundation of floodplains and dambos due to unusually heavy rainfall, allowing a large number of infected *Aedes* eggs to hatch, like in Kenya [5] or following the introduction of infected vectors or animals in flooded areas as hypothesized in Saudi Arabia and Yemen [6]. Animals are infected via bites from infectious vectors, and the sustainability of local transmission is supported by the presence of more permanent bodies of water in the environment which creates suitable conditions for *Culex* mosquitoes to breed and act as secondary vectors [7]–[11]. The extent of virus spread in time and space during RVF epidemics is believed to be attributed to active or passive vector dispersal, but also to the movements of infectious animals, either wild or domestic [12]. Although practically challenging to study because of data scarcity, knowledge on the relative importance of vector dispersal versus movements of infectious animals would be useful to inform disease control. For infectious diseases, the presence of space-time interaction between cases, which is the extent to which cases are spatially and temporally proximate, can be interpreted as an indicator of an underlying contagious process [13]–[17]; and measuring and quantifying it may assist in generating hypotheses on the different mechanisms of transmission involved in disease spread. The analysis of space-time interactions using the space-time *K*-function, has previously been explored for a variety of animal infectious diseases, such as sheep scab [18], foot-and-mouth disease [19], [20] and equine grass sickness in Great Britain [21]; tuberculosis in cattle farms in New Zealand [22], infectious bursal disease in broilers in Denmark [23], and recently foot-and-mouth disease in Tanzania [24] and porcine high fever disease in Viet Nam [25].

In South Africa, three major country-wide epidemics occurred in 1950–1951 [26], in 1973–1975 [27] and lately in 2008–2011. As of April 2012, very few descriptions of these epidemics have been published [26]–[29]. This paper presents a first step to improve our understanding of the space-time pattern of RVF in South Africa using the 2008–2011 dataset collated from World Animal Health Information Database [30]–[34]. During these four years, a total of 690 farms were confirmed RVF positive. About 95% (n = 658) of the farms contained the most susceptible species to RVF infection, that is, domestic livestock including cattle, small ruminants (sheep or goats) or both; the remaining farms raising Camelidae or wild animals. In the present paper, we used the RVF domestic livestock data subset to describe the spatial and temporal pattern of RVF in 2008–2011, and, by using the space-time *K*-function, to quantify the presence of a potential transmission process, in order to generate hypotheses on the different mechanisms of RVF transmission.

## Methods

### Data and case definition

The dataset contained 658 RVF cases, defined as reports from farms raising only cattle, small ruminants (sheep or goats), or both, in South Africa, between 2008 and 2011, collated from the World Animal Health Information Database [30]–[34]. Available information comprised the GPS coordinates of the affected farms, the starting date of the outbreak (day precision), the host species, and where available the number of susceptible animals, cases and animal deaths on the farm. Since RVF is an “OIE (World Organisation for Animal Health) Listed Disease”, diagnosis was made using standardised RVF diagnostic tests [35].

### Descriptive analysis

Epidemic curves showing the daily number of cases for the years 2008, 2009, 2010 and 2011 were produced, and cases were mapped. Descriptive on-farm statistics were calculated, including on-farm morbidity and case fatality proportions. On-farm morbidity was obtained by dividing the number of cases by the number of susceptible animals present on farm; and case fatality was the number of deaths divided by the number of cases.

### Spatiotemporal analyses

Space-time interaction was investigated using the space-time *K*-function, *K(s,t)*, defined as the expected number of cases that occur within separating distance *s* and time *t* of a previously randomly selected case, divided by the mean number of cases per unit space per unit time, also termed “intensity” [14]. In the absence of space-time interaction, that is, when cases occur independently in time and space, *K(s,t)* is the product of two *K*-functions in space *K*_1_(*s*) and in time *K*_2_(*t*); such as: *K* (*s,t*) = *K*_1_(*s*) *K*_2_(*t*) (Eq 1). If we define *D*(*s,t*) the difference *D*(*s,t*) = *K*(*s,t*)−*K*_1_(*s*) *K*_2_(*t*) (Eq 2), then positive values of *D*(*s,t*) indicate the presence of space-time interaction; and the higher *D*(*s,t*), the stronger the evidence. Because *D(s,t)* naturally increases with space and time, we calculate *D_0_*(*s,t*) = *D*(*s,t*)/*K_1_*(*s*) *K_2_*(*t*) (Eq 3), which is the number of events attributable to space-time interaction divided by the number of events in the absence of a space-time interaction. *D_0_*(*s,t*) is therefore interpreted as the proportional increase, or excess risk, attributable to the space-time interaction [14]. *D_0_*(*s,t*)>1 indicates that the number of observed events was greater than twice the number of expected events [23]. Under the null hypothesis of no space-time interaction, the dates of case reports are randomly permuted on the fixed set of case locations using Monte Carlo simulation, therefore generating a distribution for *D*(*s,t*) values. This distribution is compared with the *D*(*s,t*) calculated from the observed data, and if it exceeds 95 percent of the simulated *D*(*s,t*) values, then it can be concluded that there is less than 5% probability that the observed space-time interaction occurred by chance [17], [36].

The space-time *K*-function was calculated separately for the years 2008, 2009 (for each distinct wave), 2010 and 2011. Maximum separation distances of 300 km and 60 days were used for *s* and *t* dimensions to investigate long-distance transmission mechanisms, and to allow farms' infectiousness to persist twice as long as the 30 days assumed at the animal level by the OIE [35]. *D*(*s,t*) was estimated from 999 Monte Carlo random date permutations. The analysis was implemented using the splancs library [37] from the statistical package R version 2.14.0 [38].

## Results

### Descriptive analysis

**Figure 1:**
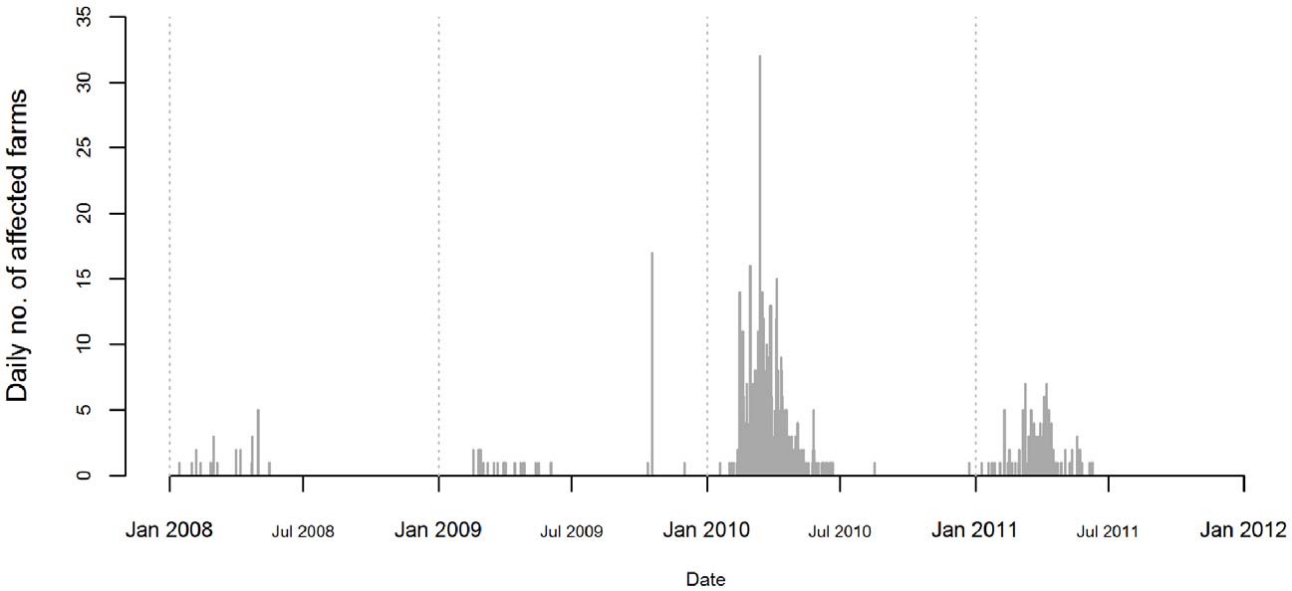
Daily number of RVF affected farms in South Africa, between 2008 and 2011.

**Figure 2:**
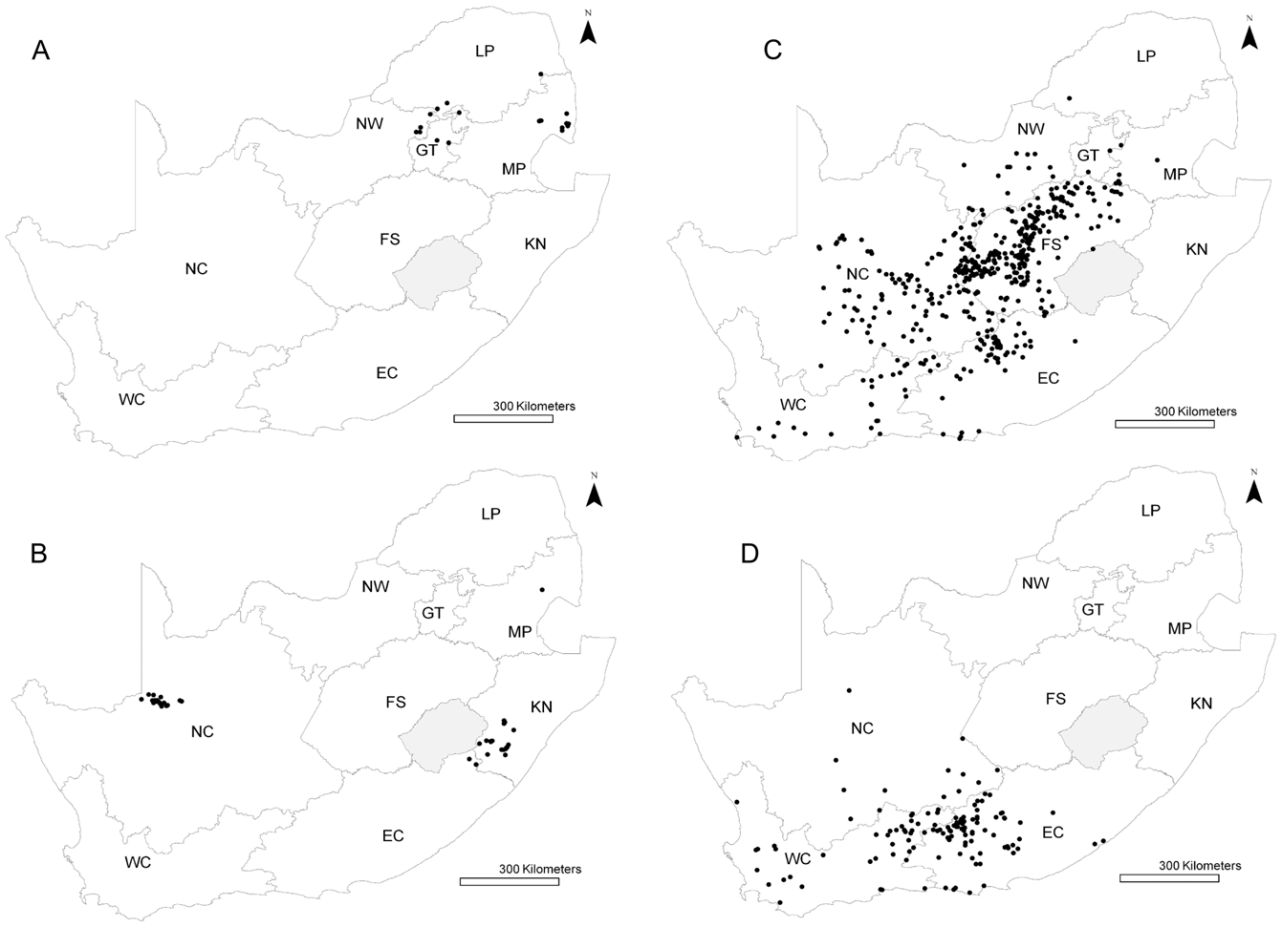
RVF livestock cases for the years 2008 **(A), 2009 (B), 2010 (C) and 2011 (D).** For 2009, both waves are displayed. Provinces are NC: Northern Cape, WC: Western Cape, EC: Eastern Cape, FS: Free State, NW: North West, KN: KwaZulu-Natal, MP: Mpumalanga, GT: Gauteng, LP: Limpopo. The light gray shaded area is Lesotho (no data).

Between 2008 and 2011, 658 RVF cases were reported in five distinct waves of varying size and location. Over 70% (n = 471) of the cases were reported in 2010 (Table 1). The occurrence of RVF was seasonal, with most cases occurring between January and April, and reported until July (Figure 1); except in 2009 when RVF cases resumed in October. Figure 2 shows the spatial distribution of RVF cases reported during the period 2008–2011. In 2008, Mpumalanga, North West, Gauteng and Limpopo provinces were affected (Figure 2A). In 2009, cases from the first wave were located in the east of the country, mostly in KwaZulu-Natal province; and the second wave occurred in the Northern Cape, near the Namibian border (Figure 2B). In 2010, the epidemic was almost country-wide, except for the eastern low-lying areas (Figure 2C). Finally, in 2011, cases were mostly distributed in southern South Africa, mainly in the Western Cape and Eastern Cape provinces (Figure 2D).

**Table 1:** Number of affected farms (%) per outbreak wave, by on-farm species.

	Number of affected farms (%)					
On-farm species	2008	2009, wave 1	2009, wave 2	2010	2011	All years
CA	21 (87.5)	18 (90.0)	6 (31.6)	62 (13.2)	19 (15.3)	126 (19.1)
SR	3 (12.5)	2 (10.0)	3 (15.8)	232 (49.3)	100 (80.6)	340 (51.7)
SR+CA	-	-	10 (52.6)	177 (37.6)	5 (4.0)	192 (29.2)
Total per year (100%)	24	20	19	471	124	658

SR = small ruminants, CA = cattle.

Across the four years, the mean on-farm morbidity varied from 0.02 to 0.23 in 2008–2009, 0.07 to 0.09 in 2010, and 0.07 to 0.21 in 2011. The mean on-farm case fatality ranged from 0.29 to 1.00 in 2008–2009, 0.66 to 0.79 in 2010, and 0.85 to 1.00 in 2011 (Table 2). Finally, for the four years, the mean morbidity and case fatality proportions for cattle farms were 0.08 and 0.74 respectively; 0.10 and 0.81 for small ruminant farms, and finally 0.07 and 0.67 for farms raising both (Table 2).

**Table 2:** Number of farms affected by Rift Valley fever in 2008, 2009, 2010 and 2011, raising cattle, small ruminants or both.

		On-farm morbidity		On-farm case fatality	
Species on farm	No. Affected farms (%)	Mean (sd)	Sample size	Mean (sd)	Sample size
2008					
CA	21 (87.5)	0.18 (0.20)	10	0.62 (0.46)	21
SR	3 (12.5)	0.19 (0.15)	2	0.56 (0.07)	3
Total	24 (100)	0.18 (0.19)	12	0.61 (0.43)	24
2009, wave 1					
CA	18 (90)	0.02 (0.03)	16	0.60 (0.42)	17
SR	2 (10)	0.09 (n.a.)	1	0.50 (0.71)	2
Total	20 (100)	0.02 (0.03)	17	0.59 (0.43)	19
2009, wave 2					
CA	6 (31.6)	0.23 (0.22)	6	0.29 (0.28)	5
SR	3 (15.8)	0.07 (0.05)	3	1.00 (0.00)	3
SR+CA	10 (52.7)	0.03 (0.04)	8	0.62 (0.49)	7
Total	19 (100)	0.10 (0.16)	17	0.58 (0.44)	15
2010					
CA	62 (13.2)	0.07 (0.10)	54	0.79 (0.35)	61
SR	232 (49.3)	0.09 (0.19)	205	0.79 (0.32)	228
SR+CA	177 (37.6)	0.07 (0.10)	170	0.66 (0.35)	174
Total	471 (100)	0.08 (0.15)	429	0.74 (0.34)	463
2011					
CA	19 (15.3)	0.07 (0.23)	19	0.94 (0.23)	19
SR	100 (80.6)	0.11 (0.21)	97	0.85 (0.30)	100
SR+CA	5 (4.00)	0.21 (0.44)	5	1.00 (0.00)	5
Total	124 (100)	0.10 (0.22)	121	0.87 (0.29)	124
All years					
CA	126 (19.1)	0.08 (0.15)	105	0.74 (0.39)	123
SR	340 (51.7)	0.10 (0.20)	308	0.81 (0.31)	336
SR+CA	192 (29.2)	0.07 (0.12)	183	0.67 (0.35)	186
Total	658 (100)	0.09 (0.17)	596	0.76 (0.34)	645

For each type of farm, on-farm morbidity and fatality are provided.

CA = cattle, SR = small ruminants, sd = standard deviation.

### Spatio-temporal analyses

**Figure 3:**
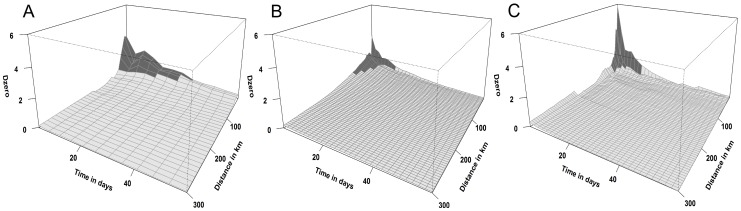
Plot of excess risk attributed to the space-time interactions (*D_0_*(*s,t*)) showing the spatial (distance in km) and temporal (time in days) distances at which clustering occurred in 2009 (wave 1) **(A), 2010 (B) and 2011 (C).** The white shaded areas show the space-time distances for which *D_0_*(*s,t*)>1.

Table 3 presents the spatiotemporal distances at which an excess risk (*D_o_*(*s,t*)>1) was attributable to space-time interaction, together with their *p*-values. No space-time interaction was present during the second 2009 wave and only weak evidence was found in 2008 (*p*-value = 0.091, Table 3). *D_o_*(*s,t*) plots were produced for the waves that showed significant space-time interaction (*p*-value<0.05), that is, the first 2009 wave and the 2010 and 2011 ones (Figures 3A, 3B and 3C). Detailed examination of the *D_o_*(*s,t*) values for 2009 showed evidence of a short (1 day) and intense contagious process (excess risk >3) up to 20 km. The intensity of the space-time interaction decreased but remained for a month, up to 40 km (Table 3 and Figure 3A). Initial and localised transmission processes were observed in the 2010 and 2011 waves (3 days over 5 km and 3 days over 15 km, respectively), although the intensity of the transmission seemed to be more important in 2011 (maximum excess risk = 5.88) compared with 2010 (maximum excess risk = 3.20). However, although reduced (1<*D_o_*(*s,t*)<2), the spatial extent of the transmission was almost 3 times more important in 2010 (90 km) than in 2011 (35 km) within the same time-window of 13 days (Table 3, Figures 3B and 3C).

**Table 3:** Excess risk attributed to the space-time interactions (*D_o_*(*s,t*)), and corresponding *p*-values, by wave.

	Separating distances		Results			
Year (wave)	Time (60 days)	Space (300 km)	*D_o_*(*s,t*)	Upper time window	Upper space window	*p*-value
2008	2 days	5 km	>2	9 days	15 km	0.091
			>1	35 days	50 km	
2009 (1)	5 days	10 km	>3	1 day	20 km	0.008
			>2	11 days	30 km	
			>1	31 days	40 km	
2009 (2)	5 days	10 km	>2	-	-	n.a.*
			>1	-	-	
2010	2 days	5 km	>3	1 day	5 km	<0.001
			>2	3 days	5 km	
			>1	13 days	90 km	
2011	2 days	5 km	>3	3 days	15 km	0.050
			>2	5 days	20 km	
			>1	13 days	35 km	

* n.a.: not applicable: *D_o_*(*s,t*) values were below unity.

## Discussion

Rift Valley fever has been reported in South Africa over the last four years, showing a seasonal pattern mainly between January and July. About 70% of the cases reported between 2008 and 2011 occurred in 2010. Each year, a different part of the country has been affected, with the 2010 epidemic being almost country-wide. In other years, cases were confined to a few provinces. No strong evidence of space-time interaction was found in 2008 and in the second wave in 2009. In the first wave of 2009, a significant space-time interaction was detected for up to one month and over 40 km. In 2010 and 2011 a significant intense, short and localised space-time interaction (up to 3 days and 15 km) was detected, followed by one of lower intensity (up to 2 weeks and 35 to 90 km).

The season between January and April (mid-summer to autumn), brings rain in most parts of the country, and corresponds to the period when *Culex theileri*, *Aedes juppi, Aedes mcintoshi* and other members of the *Aedes (Neomelaniconion)* genus, the main RVF epidemic vectors in South Africa, are the most prevalent mosquitoes [39]. Our results, showing significant contagious processes during these seasons for the years 2009, 2010 and 2011, are in line with the hypothesis that mosquito bites are the principal infection mechanism of RVF in South Africa. While these results are to be expected for a vector-borne disease, the absence of contagious process in 2008 and the second 2009 wave, and the various extents and intensities of the space-time interactions found across the different years could support further evidence that other transmission mechanisms may also exist.

Active dispersal for most RVF vectors is short, and although little information is available, it is estimated to be about 1 km, varying from less than 150 m for *Aedes* to approximately 2 km for *Culex theileri* [12], [40]. In addition, the analysis of spatial and space-time clusters for dengue, a human disease mainly transmitted by *Aedes aegypti*, showed a local transmission varying between 800 m and 4 km [41]–[43], and spatio-temporal clusters over short distances from 400 m to 2.8 km, sustained over 2 to 13 weeks [41], [44], [45]. These vector-borne transmission patterns share some similarities with the initial and localised contagious processes observed during RVF epidemics in 2010 and 2011, but our study detected the presence of an additional spatiotemporal process, with RVF potentially spreading to distances up to 40 to 90 km, within about 2 weeks. This appearance of long-distance spread could be explained by the existence of several RVF virus emergences; defined as distinct hatchings of infected *Aedes* eggs or multiple re-introductions of infected vectors from areas external to our study area. However, similar extended spatio-temporal patterns as those observed in this study have been described for foot-and-mouth disease in Tanzania, reaching 50 km to hundreds of kilometres over a 2 week period [24] and for avian influenza in Bangladesh up to 150–300 km [46]; both diseases for which the movements of animals were likely to play a major mechanism of spread [47]–[49], [50]. Therefore, this suggests that RVF spread over distances larger than the assumed range of active vector dispersal could be explained by the movement of domestic or wild viraemic and therefore infectious animals. Other mechanisms of long-distance spread could also be incriminated, such as wind-borne vector dispersal, which has been described up to 100 km for some *Aedes* and *Culex* species [40]. Finally, in early 2009 in KwaZulu-Natal province, space-time interaction was present up to 20 km within 1 day. Such a pattern probably allowed ruling out active vector dispersal in favour of animal movements, or multiple local emergences.

Several limitations in these analyses may have affected our results and their interpretation. Firstly, this study relies on RVF cases that were reported to the World Organisation for Animal Health (OIE) and are likely to represent only a subset of the total number of infected farms in South Africa. From a statistical perspective, the type I error of the space-time *K*-function has been shown to remain low with under-reporting of cases [51], which means we can be confident that the space-time interactions found in 2009, 2010 and 2011 actually existed. Also, Fenton et al. 2004 [51] showed that the *K*-estimate was a good reflection of the underlying contagious process, when the probability of a farm not being reported increased proportionally with increasing distances from a random point, assumed to be a regional laboratory centre, which is likely to be the case for a notifiable disease. However, the study power, i.e. the ability of the test to detect a space-time interaction when there is one, was more dependent on sample size [51], which makes it difficult to know whether the absence of space-time interaction in 2008 was likely to be true or resulted from the small number of reported positive farms. While no published outbreak investigation has been identified for this 2008 outbreak, Anyamba et al 2010 [52] reported that the current early warning system, based on climatic factors, forecasted suitable conditions for virus re-emergence on a regional scale (Southern Africa) in February 2008. However, no larger epidemic followed, suggesting an absence of suitable environmental conditions for producing significant populations of secondary vectors to amplify the virus to epidemic proportions.

The absence of contagiousness for the second wave of 2009 is easily explained by the fact that 89% (17/19) of the cases were reported on the same day (October 19, 2009). If cases truly occurred in different locations on the same day, this would suggest that the virus was evenly distributed in the environment and emerged at the same time. For this wave, one outbreak investigation was published [53]; reporting no abnormal climatic conditions that could explain high mosquito densities, but hypothesized flood irrigation techniques as a factor for virus emergence, and a low number of animals precluding to sustain an epidemic. In addition, recent genome sequencing revealed that RVF viruses from the same lineage H caused the outbreaks in Namibia in 2004, these late 2009 cases, and the 2010/2011 ones in South Africa, suggesting an epidemiological link between them [54].

Secondly, the definition of the space-time *K*-function is based on several assumptions that may have affected our results. For example, the space-time *K*-function assumes that the underlying first-order effects are constant across the space-time study environment [14], [15], [17], therefore considering that all cases arose from second-order effects. In our study, this means that cases within 300 km and 60 days of any arbitrary case were treated as if resulting from transmission only and none were due to emergence. Since the existence of multiple foci of RVF virus emergence cannot be totally excluded, by artificially decreasing the number of potential ‘parent cases’ in the dataset, that is RVF foci, we tended to overestimate the study power [51]. Further environmental data would be necessary to identify potential RVF foci resulting from *Aedes* hatching, although infected farms located in such suitable environment could also have been infected by transmission from neighbouring infected farms. Another assumption of the space-time *K*-function is that the density of the population at risk does not vary, or varies evenly over time [13]–[15], [17]. In practice, the population at risk is likely to have reduced over time due to animal vaccination or life-long immunity induced by natural infections [4], and to culling procedures that removed previously diagnosed animals. The timing and location in which these activities (i.e. vaccination and culling) were implemented are both difficult to estimate since they depended on farmers' decisions. However, a decrease in the number of susceptible farms over time would have resulted in under-estimating the intensity of the space-time interaction, which makes our results conservative.

Thirdly, it is acknowledged that vaccination could have been applied in some affected farms or areas during the different waves [30]–[33], but since RVF is not an officially controlled disease, vaccination coverage is not reported by the government [55]. Nevertheless, vaccination was widely advised during the 2010 epidemic [34], and it is therefore possible that some part of the areas affected in 2011 were vaccinated prior to the 2011 wave itself; leading to a possible underestimation of the *D_0_(s,t)* values.

Finally, the analysis was conducted using animal and not human cases. Whereas humans acquire infection by close contact with infected animals or their infected organs, domestic livestock are the primary hosts for RVF virus, and get infected directly from mosquito bites. Therefore the dynamics of disease in those species should better reflect vector transmission.

In conclusion, by providing a description of the spatiotemporal patterns of RVF in South Africa between 2008 and 2011, this study supports the hypothesis that during an epidemic, disease spread may be supported by factors other than active vector dispersal. To optimize disease control, these mechanisms underlying disease spread should be disentangled and quantified. This would require the use of spatiotemporal modelling tools in combination with environmental, virus genotyping, vaccination, animal movement and population at risk data.
